# Management of Kikuchi-Fujimoto Disease Using Glucocorticoid: A Case Report

**DOI:** 10.1155/2011/230840

**Published:** 2011-08-14

**Authors:** Selim Yalcin, Selami Kocak Toprak, Betul Erismis, Ozden Altundag, Handan Ozdemir, Nuray Topcuoglu

**Affiliations:** ^1^Department of Oncology, Baskent University School of Medicine, 06490 Ankara, Turkey; ^2^Department of Hematology, Baskent University School of Medicine, 06490 Ankara, Turkey; ^3^Department of Pathology, Baskent University School of Medicine, 06490 Ankara, Turkey; ^4^Department of Clinical Microbiology and Infectious Disease, Baskent University School of Medicine, 06490 Ankara, Turkey

## Abstract

Kikuchi-Fujimoto disease, also known as histiocytic necrotizing lymphadenitis, is a self-limiting, benign, and rare systemic lymphadenitis with unknown etiology. The cardinal symptoms are fever, lymphadenopathy and night sweat; consequently, it is first necessary to rule out infectious, lymphoproliferative, and connective tissue diseases such as systemic lupus erythematosus. Histology can allow diagnosis by demonstrating necrotizing histiocyte lymphadenitis. Disease, which has no specific treatment, self-limits itself in 1 to 6 months clinically. However, non-steroid anti-inflammatory agents can be given for symptomatic treatment and there are reports using corticosteroids and antibiotics in complicated cases. This article concerns a 32-years-old female who diagnosed Kikuchi-Fujimoto disease and treated with glucocorticoid.

## 1. Introduction

Histiocytic necrotizing lymphadenitis was first described in 1972 by two independent Japanese pathologists, Kikuchi and Fujimoto [1, 2]. The classic presentation is in young women with painful cervical adenopathy and has been referred to as Kikuchi-Fujimoto disease (KFD) since the initial publications. In patients presenting with cervical adenopathy, the differential diagnosis can be broad. Here, we present the case of a young woman with KFD and review the significant features of this syndrome.

## 2. Case Report

A 32-year-old woman presented to our outpatient clinic with generalized pain, tender left cervical adenopathy, loss of appetite, night sweats, and fever to 41°C. Her recent history indicated progressive painful bilateral neck adenopathy for about two months. She had earlier presented to a local otolaryngology clinic and was given broad-spectrum antibiotics for possible lymphadenitis. She had no improvement and developed weight loss, and ultimately a biopsy was performed. Pathologic examination showed diffuse necrosis with no definite malignancy. Although the morphologic findings showed necrotizing lymphadenitis suggestive of KFD, the tissue specimen was limited, and neoplasia could not be excluded.

Her past history included appendectomy, cholecystectomy, hypertension, and levothyroxine for goiter. On physical exam, she was afebrile, and there was bilateral tender confluent cervical adenopathy without organomegaly or other significant findings. Laboratory studies were negative for toxoplasmosis, hepatitis B, hepatitis C, HIV, CMV, VDRL, and Brucella agglutination, while EBV-IgM showed a borderline positive result. Anti-dsDNA was within normal limits, while ANA, RF, angiotensin-converting enzyme, and PPD were negative. C-reactive protein (CRP) was 23.3 mg/L, and the erythrocyte sedimentation rate (ESR) was 69 mm/hr. Routine biochemical analysis, coagulation tests, and urinalysis were within normal limits. Peripheral smear showed a relative lymphocytosis. CT-neck showed bilateral cervical, submandibular, and left supraclavicular lymphadenopathy, while CT-thorax and abdomen showed no pathologic findings.

**Figure 1:**
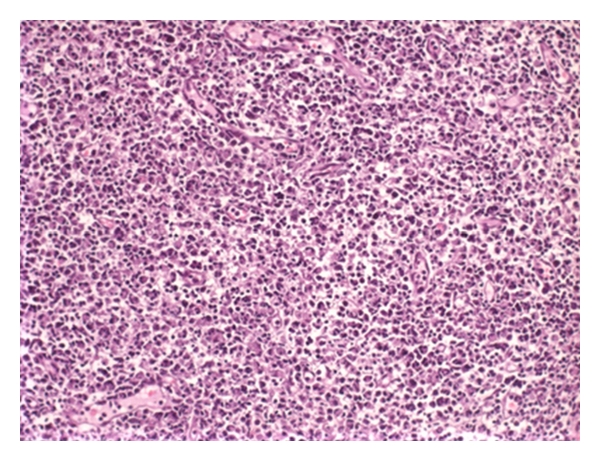
A pale staining, irregular area consists of minimal necrosis surrounded by a wide zone of immunoblasts and histiocytes (H&E, ×10).

**Figure 2:**
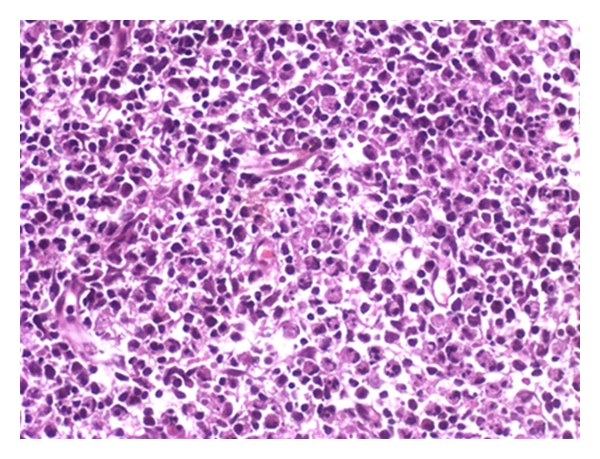
Necrotic area devoid of polymorphonuclear leukocytes but contains karyorrhectic debris (H&E, ×40).

**Figure 3:**
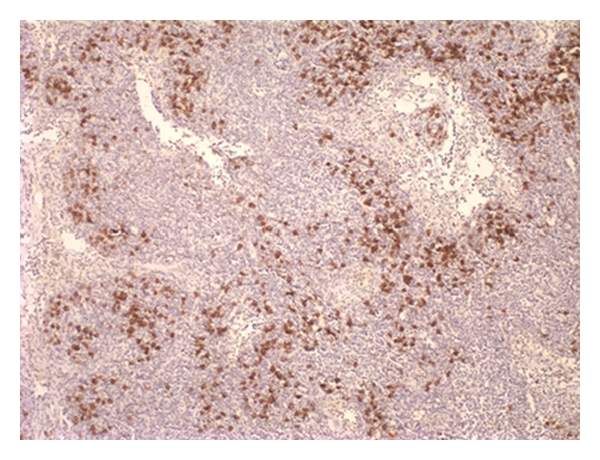
Numerous CD68 histiocytes surrounding the areas consist of karyorrhectic debris (H&E, ×10).

With the potential diagnosis of KFD, nonsteroidal anti-inflammatory drug (NSAID) therapy with meloxicam and diclofenac was given along with ciprofloxacin. She had persistent fever and received additional antibiotics, blood cultures eventually grew methicillin-resistant coagulase-negative Staphylococcus, and teicoplanin therapy was started. Repeat cervical node excision was performed, revealing lymphadenitis with predominant histiocyte and plasmacytoid-like lymphocytes along with necrotic debris. This was felt to be consistent with KFD (Figures 1, 2, and 3). 

During this period, there was no significant improvement in her clinical course, despite antibiotic and NSAID therapy, so methylprednisolone at 56 mg/day (1 mg/kg) was started. She received this dose for 8 days, and then the dose was tapered 8 mg in every consecutive three days. Her temperature returned to normal. Following the regulation of corticosteroid treatment (8 mg/day, po), the patient was scheduled for close followup and discharged. At next followup in one month, lymphocytosis had regressed; CRP and ESR had returned to the normal range, and cervical adenopathy was decreased by about 50%. Two months later, physical examination and laboratory measurements were within normal limits, and it was decided to discontinue corticosteroid therapy (8 mg/day, po). The patient is currently not receiving any treatment after 3 months of followup and is undergoing further assessment for possible connective tissue disorder.

## 3. Discussion

KFD is a syndrome of unknown etiology. It is generally associated with fever, sweats, and progressive painful adenopathy, primarily in the cervical region. The disease is rare, and most reports are from the Asian countries, especially Japan [3]. Most patients are under the age of 30, with a female-to-male ratio of 4 : 1 [4].

There are many speculations as to etiology with both viral and autoimmune processes suspected. Human Herpesvirus 6, Human Herpesvirus 8, parvovirus B-19, and Epstein-Barr virus have all been implicated. The presentation is generally acute, with progression over a 2-3 week period. In this time frame, painful cervical adenopathy dominates, with generalized lymphadenopathy uncommon [4], and fever sometimes exceeds 40°C. Other symptoms include night sweats, upper respiratory complaints, and weight loss. Laboratory analyses generally reveal leucopenia with a relative lymphocytosis.

The diagnosis depends on examination of an excisional biopsy with characteristic histopathologic findings of KFD, including paracortical areas of coagulative necrosis with abundant karyorrhectic debris, distortion of the nodal architecture, and large numbers of histiocytes at the margins of necrotic areas [4]. Although uncommon, the diagnosis of KFD should be considered in a differential diagnosis that includes tuberculosis, connective tissue diseases such as SLE, or lymphoproliferative disorders. Indefinite clinical followup of KFD has been suggested for emergence of SLE or other connective tissue disorders.

The process is typically limited from one to six months [4], with recurrence reported in 3-4% of patients [5]. Lymphadenopathy can resolve spontaneously. Following definitive diagnosis, the essentials of treatment include analgesia, antipyretics, and NSAID therapy. Although rare, if extracervical or extranodal disease is present, corticosteroid treatment is recommended [4].
